# Carbapenem-Resistant Enterobacteriaceae: A Retrospective Review of Presentation, Treatment, and Clinical Outcomes in a Tertiary Care Referral Hospital

**DOI:** 10.7759/cureus.27094

**Published:** 2022-07-21

**Authors:** Mohammed Al Khamis, Zainab AlMusa, Mai Hashhoush, Narjis Alsaif, Abdul Salam, Manal Atta

**Affiliations:** 1 Critical Care Department, King Fahad Specialist Hospital, Dammam, SAU; 2 Internal Medicine/Infectious Diseases, King Fahad Specialist Hospital, Dammam, SAU; 3 Department of Pharmacy, King Fahad Specialist Hospital, Dammam, SAU; 4 Department of Epidemiology and Biostatistics, King Fahad Specialist Hospital, Dammam, SAU; 5 Department of Laboratory, King Fahad Specialist Hospital, Dammam, SAU

**Keywords:** saudi, mortality, intensive care, risk factors, carbapenem-resistant enterobacteriaceae

## Abstract

Background: Carbapenem-resistant Enterobacteriaceae (CRE) is an emerging infectious threat with an increasing incidence locally and worldwide. It carries a high morbidity and mortality; focusing on this topic should be a priority in clinical research as local data are not widely available. The objective of this study is to describe the presentation, risk factors, treatment pattern and clinical outcomes associated with CRE infections.

Methods: We conducted a cross-sectional retrospective study in a single center tertiary referral hospital. We included adult patients above 18 years of age with infection due to CRE between January 1, 2018, to December 30, 2019.

Results: During this period, 76 cases were studied. The mean age of the cases was 54 years. The majority were immunosuppressed and admitted to the intensive care unit. The most frequent risk factors associated with CRE infection among study subjects included prior antibiotics in the preceding three months and prior hospital admission in the last one year. Klebsiella pneumoniae (77%) represented the most isolated organism. All-cause intensive care unit and in-hospital mortality were significantly higher among patients with pneumonia and bacteremia.

Conclusions: CRE infections are associated with higher morbidity and mortality specifically in patients who presented with pneumonia and bacteremia. High resistance rate and limited treatment options have made a great variability in the clinical practice. Appropriate definitive treatment of CRE infections, strict infection control measures, and antimicrobial stewardship program activation are essential.

## Introduction

Carbapenem-resistant Enterobacteriaceae (CRE) is an emerging problematic infectious pathogen, with reports of its prevalence worldwide [1]. An increase in carbapenem consumption as a result of the global spread of extended-spectrum b-lactamase (ESBLs) has increased the selection pressure and facilitated the spread of CRE [2]. The reported incidence of CRE infection in the United States is 2.93/100,000 people [3,4]. In a recent systematic review, the prevalence of CRE in Saudi Arabia has been reported to be between 12% to 32% [5]. High mortality rates, ranging from 30% to 75%, have been reported for patients with severe CRE infections [2,6-8].

Several risk factors for CRE acquisition have been reported in the literature; these include but are not limited to: admission to an intensive care unit (ICU), longer hospital and ICU stay, prior hospitalization, invasive devices, mechanical ventilation, steroid use, transplant recipient, parenteral nutrition and prior antimicrobial therapy particularly carbapenem [9,10]. Rectal colonization with CRE has been invariably identified as a risk factor for the development of subsequent CRE infection. In a recent meta-analysis, approximately 16.5% of CRE colonized cases developed infection [6]; another observational trial with a small number of patients included only ICU patients and reported a lower rate of CRE infection of 8% in the subsequent 30 days following the rectal screening [11].

There are concerns about other classes of antibiotics commonly used to treat CRE infections such as colistin, tigecycline, and fosfomycin. There is insufficient data on their effectiveness, as well as limited clinical experience with their use, more frequent adverse effects, and rapid development of resistance during treatment which make treating those infections a real challenge [12].

The primary objective of this study was to describe the presentation of CRE-related infections in our cohort of patients, associated risk factors, treatment approach, and their clinical outcomes. This will help as a quality metric and will be a source of information that might help future researchers in the region.

## Materials and methods

Setting and study design

We conducted a retrospective analysis of patients with CRE infection at a single tertiary center, King Fahad Specialist Hospital Dammam, Saudi Arabia, after having IRB approval for the study (approval number ICU0305). The hospital mainly provides transplant, oncology, and neuroscience services. Patients were identified from the microbiology lab if CRE pathogens were identified from respiratory, blood, urine, bile, superficial or deep pus, or sterile body fluid samples from January 1, 2018, to December 30, 2019, and then patient’s medical records were reviewed retrospectively. Any patient above the age of 18 who had a positive culture for CRE and a documented source of infection requiring antimicrobial treatment was eligible for inclusion. Patients deemed to have CRE colonization were excluded. If the patient developed another episode of CRE infection in the same admission or was readmitted to the hospital during the study period, we recorded the first episode. Patients’ data were compiled until death or hospital discharge. The hospital is conducting susceptibility of Enterobacteriaceae according to Clinical and Laboratory Standards Institute (CLSI) breakpoint definitions. Carbapenem resistance was defined according to Performance Standards for Antimicrobial Susceptibility Testing M100; 29th Edition 2019 [13]. Enterobacterales isolates were included in our study based on the phenotypic identification of isolates by the commercial identification Vitek 2 system (bioMérieux, Marcy-l'Étoile, France) using the most recent edition of the CLSI document M100 that provides the carbapenem zone diameter and minimum inhibitory concentration (MIC) breakpoint guidelines for Enterobacterales [13]. A multiplex polymerase chain reaction (PCR) was used for detection of carbapenems resistance genes (IMP, VIM, KPC, NDM-1 and OXA-48) using the Xpert Carba-R assay (Cepheid, Sunnyvale, CA, USA). This test was validated to identify these genes for the Enterobacterales isolates phenotypically identified as CRE by the Vitek 2 system.

Clinical definitions

We used the CDC definition of CRE [14]. Escherichia coli, Klebsiella pneumoniae and Enterobacter species are defined as CRE if not susceptible to one of the carbapenems; for Morganella morganii, Proteus species and Providencia species as these organisms have intrinsically elevated MICs to imipenem, it requires non-susceptibility to carbapenems other than imipenem to be considered as CRE. Colonization is defined as a culture of CRE obtained from any site within 90 days before the qualifying CRE infection or if the patient has a rectal swab positive for CRE. Sepsis or septic shock is defined according to the Third International Consensus Definitions for Sepsis and Septic Shock (Sepsis-3) [15], and is recorded when developed 48 hours before or after the culture. If the organism is sensitive, it will be labelled as susceptible. If intermediate or resistant it will be labelled as non-susceptible. Immunosuppressed patients included those with active malignancy receiving chemotherapy or radiotherapy, transplant patients on immunosuppressive medications, or those on systemic steroids greater than 0.3 mcg/kg/day of prednisone or its equivalent daily for a period longer than two weeks. Empiric therapy was defined as any therapy given within 72 hours of the culture and before the availability of the susceptibility results. Directed therapy was defined as any therapy given based on the culture and susceptibility results. All-cause ICU and hospital mortality were recorded if mortality happened at any time during the defined study period.

Statistical analyses

Descriptive and inferential statistics were used to characterize the study sample and test hypotheses. Descriptive results for all continuous variables are presented as mean ± standard deviation (SD; for normally distributed data), or median with inter-quartile range (for data not normally distributed), while numbers (percentage) are reported for all categorical variables. Bivariate analyses were performed using Pearson Chi-Square test or Fisher Exact test whenever appropriate to assess the relationship between demographic and clinical characteristics with ICU and hospital mortality. Simple and multiple logistic regression analyses were also performed to identify factors that are associated with ICU and hospital mortality after adjusting for potential confounders. Odds ratio (OR), adjusted odds ratio (AOR), and 95% confidence interval (CI) were reported. A P value <0.05 (two-tailed) was considered statistically significant. All statistical analyses were performed using the Statistical Package for Social Sciences (SPSS) version 28 (IBM Corp., Armonk, NY, USA).

## Results

**Figure 1:**
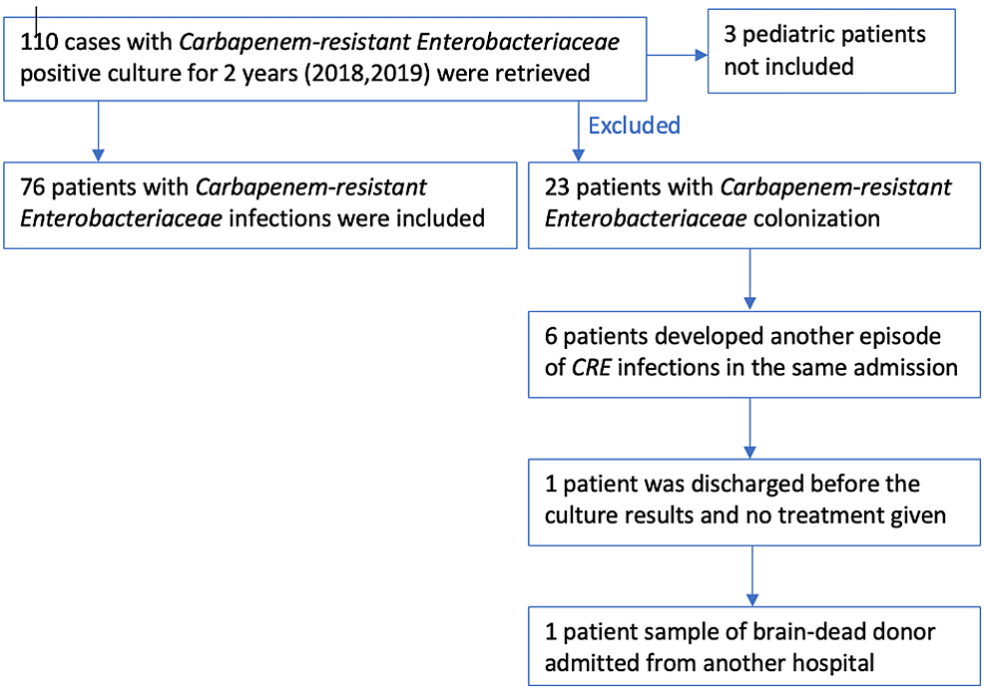
Study identification, screening, and eligibility

Four thousand, nine hundred and twenty-two patients with Enterobacteracia-positive cultures were identified during the period from January 1, 2018, through December 31, 2019, from the microbiology database; out of them, 110 patients were found to haveCRE (2.2%). Seventy-six patients with CRE infections were included and 34 patients were excluded (Figure 1). The demographic characteristics of included patients are detailed in Table 1. The majority were diabetic and immunosuppressed with solid malignancy or post-organ or bone marrow transplant. The most frequent source of infection among the study subjects was bacteremia followed by pneumonia, 46% of the patients were admitted from the emergency room, 23% had elective admission, and 60.5% were admitted to ICU: 26 (34%) had septic shock and 14 (18%) had sepsis.

**Table 1:** Baseline characteristics and comorbidities of included patients (N=76) *Results are expressed as mean ± SD for age and number (percentage) for other factors. DM: diabetes mellitus, CKD: chronic kidney disease, CHF: congestive heart failure

Factors	Results
Age (years)	54.0 (18.3)
Gender	
Male	39 (51%)
Female	37 (49%)
Co-morbidities	
DM	35 (46%)
Hemiplegia	6 (8%)
On immunosuppressive treatment	24 (32%)
CKD	12 (16%)
CHF	5 (6.6%)
Solid organ transplant	10 (13%)
Bone marrow transplant	10 (13%)
Liver disease	10 (13%)
Solid Tumor	25 (33%)
Hematologic malignancy	5 (6.6%)
Ischemic heart disease	7 (9%)
Peripheral vascular disease	5 (6.6%)
Source of infection	
Bacteremia	21 (28%)
Intra-abdominal Infection	13 (17%)
Pneumonia	19 (25%)
Skin/soft tissue infection	10 (13%)
Urinary tract infection	17 (22%)
Surgical wound infection	9 (12%)
Source of admission	
Another hospital	21 (28%)
Elective admission	17 (23%)
Emergency room	33 (46%)
OPD	5 (7%)
Type of patient	
Hematology	6 (8%)
Medical	13 (17%)
Other	11 (14%)
Oncology	17 (22%)
Surgical	19 (25%)
Transplantation	10 (13%)
ICU admission	
Admitted to ICU	46 (60.5%)
Not admitted to ICU	30 (39.5%)
Sepsis/septic shock	
Sepsis	14 (18%)
Septic shock	26 (34%)
Active infections no sepsis	36 (48%)

The median Charlson Comorbidity Index (CCI) for the study subjects was 4. The median days on mechanical ventilation and vasoactive medications for those admitted to ICU were seven and three, respectively. The median for the total ICU and hospital length of stay was 11 and 31.5, respectively (Table 2). 

**Table 2:** Clinical characteristics of included patients *Results are expressed as median (IQR). LOS: length of stay, CRE: carbapenem-resistant Enterobacteriaceae

Factors	Results
Charlson Comorbidity Index (CCI)	4 (2 - 7)
Days on Mechanical Ventilation ^(N=46)^	7 (2 - 31)
Days of vasoactive medications ^(N=46)^	3 (2 - 8)
ICU length of stay before CRE (days)	0 (0 - 7)
ICU length of stay (total day during the index infection episodes) ^(N=46)^	11 (4 - 36)
Hospital LOS before CRE (days)	10 (0 - 24.5)
Hospital LOS CRE total (days) ^(N=72)^	31.5 (18 - 53.25)

The most frequent risk factors for CRE acquisition among study subjects included prior antibiotics in the preceding three months (95%), prior hospital admission in the last one year (91%), followed by the presence of invasive devices (33%) (Table 3).

**Table 3:** Risk factors associated with CRE infection *Results are expressed as number and percentage. CRE: carbapenem-resistant Enterobacteriaceae

Factors	Results
Prior Antibiotic in the preceding 3 months	72 (95%)
Prior hospital admission in the last 1 year	69 (91%)
Invasive devices	25 (33%)
Lack of source control	14 (18%)
Prior CRE colonization Infection up to 1 year	10 (13%)

K. pneumoniae (77%) represented the most isolated organism followed by E. coli (17%). Molecular analysis was performed on only 12 isolates: OXA-48 (10 samples), NDM (one sample), and one isolate had both NDM and VIM (Table 4).

**Table 4:** Species of CRE isolated *Results are expressed as number and percentage. CRE: carbapenem-resistant Enterobacteriaceae

Species	Results
K. pneumoniae	59 (77%)
E. coli	13 (17%)
Proteus	2 (3%)
Enterobacter cloacae	1 (1%)
Serratia	1 (1%)
Total	76 (100%)

Combination empirical antimicrobials were prescribed to 53 (70%) of the patients while a single antimicrobial was prescribed for 23 (30%) of the patients. The most frequently prescribed empirical antimicrobials for the patient cases either single or in combination were piperacillin/tazobactam (28%), imipenem/cilastatin (26%), meropenem (25%), colistin (16%) and ciprofloxacin (10%); while for targeted therapy, tigecycline (37%) colistin (28%), amikacin (21%), and gentamicin (11%). Susceptibility pattern of the CRE to the used targeted antimicrobials was highest with ceftazidime-avibactam and fosfomycin followed by colistin, tigecycline and amikacin, while piperacillin/tazobactam, meropenem and imipenem/cilastatin were resistant (Table 5). The all-cause mortality rate in ICU was 26% while in the hospital was 42% (Table 6).

**Table 5:** Frequency table of used empiric and targeted antimicrobial susceptibility pattern

	Empiric antimicrobial		Targeted antimicrobial	
	Susceptible	Resistance	Susceptible	Resistance
Amikacin	10	6	15	2
Tigecycline	4	1	25	2
Augmentin	0	1	0	0
Cefepime	0	3	0	1
Meropenem	0	19	0	11
Imipenem/cilastatin	0	20	0	8
Ertapenem	0	1	0	0
Piperacillin/tazobactam	0	21	0	9
Colistin	8	4	19	2
Ciprofloxacin	2	5	2	2
Gentamicin	3	0	6	2
Ceftazidime-avibactam	1	0	12	0
Ceftriaxone	0	1	0	0
Fosfomycin	0	0	1	0
Trimethoprim-sulfamethoxazole	0	0	1	0

**Table 6:** All-cause mortality rate *Results are expressed as number and percentage.

Factor	Results
ICU mortality ^(N=46)^	
Yes	20 (43%)
No	26 (57%)
Hospital Mortality ^(N=76)^	
Yes	32 (42%)
No	44 (58%)

All-cause mortality due to sepsis among the study subjects was 20% and 35.7% in ICU and in hospital, respectively, and all-cause mortality due to septic shock among the study subjects was 69.2% and 76.9% in ICU and in hospital respectively (Table 7).

**Table 7:** Hospital mortality due to sepsis/septic shock *Results are expressed as number and percentage. ¥: P-value has been calculated using Pearson

	Hospital Mortality (N=76)			ICU Mortality (N= 46)		
	No	Yes	P-value^¥^	No	Yes	P-value^¥^
			<0.001			<0.001
Active infection no sepsis	29 (80.6%)	7 (19.4%)		10 (100%)	0 (0%)	
Sepsis	9 (64.3%)	5 (35.7%)		8 (80.0%)	2 (20%)	
Septic Shock	6 (23.1%)	20 (76.9%)		8 (30.8%)	18 (69.2%)	
Total	44	32		26	20	

Most of the isolated species were resistant to prescribed empiric antimicrobials (75%). Bivariate analysis (OR) and multiple logistic regression (independent predictors, AOR) showed that bacteremia and pneumonia were significant independent positive predictors of ICU and hospital all-cause mortality. Lack of source control and receiving inappropriate empiric antibiotic has not been shown to be associated with ICU or hospital mortality (Tables 8, 9).

**Table 8:** Simple and Multiple logistic regression analysis to assess the relationship between factors (gender, risk factors, source of infection) with ICU all-cause mortality (n=46). OR: odds ratio (unadjusted); AOR: Adjusted odds ratio; CI: Confidence interval; Hosmer & Lemshow Chi-Square=1.913, p=0.928 (good fit of model to data)

	ICU mortality				
Factors	ICU mortality	OR (95% CI)	P-value	AOR (95% CI)	P-value
Gender					
Male^(n=22)^	8 (36.4%)	1		1	
Female^(n=24)^	12 (50%)	1.7(0.5-5.7)	.353	0.9 (0.2-4.3)	.97
Sensitive empirical Antibiotic					
No^(n=33)^	14 (42.4%)	1		1	
Yes^(n=13)^	6 (46.2%)	1.2(0.3-4.2)	.818	1.6(0.3-9.6)	.62
lack of source control					
No^(n=38)^	18 (47.4%)	1		1	
Yes^(n=8)^	2 (25%)	0.4(0.1-2.1)	.258	0.7 (0.1-6.7)	.73
Bacteremia					
No^(n=32)^	10 (31.3%)	1		1	
Yes ^(n=14)^	10 (71.4%)	5.5(1.4-21.8)	.015	12.9(1.8-90.8)	.01
Pneumonia					
No^(n=27)^	7 (25.9%)	1		1	
Yes^(n=19)^	13 (68.4%)	6.2(1.7-22.6)	.006	13.5(2.0-89.9)	.007
Urinary Tract Infection					
No^(n=42)^	19 (45.2%)	1		1	
Yes^(n=4)^	1 (25%)	0.4 (0.04-4.2)	.448	1.4 (0.1-25.4)	.83

**Table 9:** Simple and multiple logistic regression analysis to assess the relationship between factors (gender, risk factors, source of infection) with hospital all-cause mortality (n=76). OR: odds ratio (unadjusted); AOR: Adjusted odds ratio; CI: Confidence interval; Hosmer & Lemshow Chi-Square=6.17, p=0.628 (good fit of model to data)

	Hospital mortality				
Factors	Hospital mortality	OR (95% CI)	P-value	AOR (95% CI)	P-value
Gender					
Male^(n=39)^	17 (43.6%)	1		1	
Female^(n=37)^	15 (40.5%)	0.9 (0.36 - 2.2)	.79	0.5 (0.16 - 1.7)	.26
Sensitive empirical Antibiotic					
No^(n=55)^	24 (43.6%)	1		1	
Yes^(n=21)^	8 (38.1%)	0.8 (0.3 - 2.2)	.662	0.8 (0.2 - 3.0)	.79
lack of source control					
No^(n=62)^	28 (45.2%)	1		1	
Yes^(n=14)^	4 (28.6%)	0.5 (0.14 - 1.7)	.262	0.8 (0.2 - 3.8)	.77
Bacteremia					
No^(n=55)^	19 (34.5%)	1		1	
Yes ^(n=21)^	13 (61.9%)	3.1 (1.1 - 8.7)	.034	5.2 (1.4 - 19.2)	.014
Pneumonia					
No^(n=57)^	16 (28.1%)	1		1	
Yes^(n=19)^	16 (84.2%)	13.7 (3.5 - 53.3)	< .001>	17.3 (3.6 - 82.6)	< .001>
Urinary Tract Infection					
No^(n=59)^	28 (47.5%)	1		1	
Yes^(n=17)^	4 (23.5%)	0.34 (0.1 - 1.2)	.087	0.8 (0.2 - 3.5)	.78

## Discussion

Antimicrobial resistance is a worldwide concern and is associated with higher morbidity and mortality. In the last few years, there was a significant rise in the prevalence of multidrug-resistant organisms (MDRO) in general and CRE in particular. At our institution, the prevalence of CRE among inpatients doubled from 2.2% to 4.7% in 2018 and 2020, respectively. The rate in the ICU has also increased from 9.8% to 11.1%. 

Over 90% of our patient cohort have been exposed to at least a single antibiotic three months prior to their presentation or had been hospitalized in the last year, additionally a third of them had an invasive device present around the time of the infection. Like our study, Wang et al. [16] in a case-control study identified the previous use of third- or fourth-generation cephalosporins as an independent risk factor associated with CRE infection. Van Loon et al. [17] in a systematic review have identified 13 risk factors linked to CRE acquisition, the most was found for risk factor medical devices, followed by carbapenem use. Another study by Garbati et al. from Riyadh involving 29 cases and 58 controls of Saudi hospitalized patients identified the presence of comorbidities, prior use of carbapenems, duration of hospitalization and invasive procedures to be significantly associated with CRE infections [7].

The most common CRE isolated in our cohort was Klebsiella pneumoniae. Data from the Middle East and specifically the gulf region including Saudi Arabia is scarce [18]. Lack of reporting is probably the reason. Klebsiella pneumoniae is the most reported CRE species in Saudi Arabia with a prevalence of 88% followed by E. coli at 11% [5,19,20]. NDM and OXA-48 enzymes are the major carbapenemases causing resistance in Enterobacteriaceae reported from the Gulf region and Saudi Arabia [20-23]. Isolates possessing K. pneumoniae carbapenemase (KPC) enzymes, such as KPC-1, KPC-2, and KPC-3, are rare. Most of the CRE isolates that underwent carbapenamase gene detection in our study were harboring OXA-48 with a few producing metallo-beta-lactamase. 

The limited antimicrobial options and the lack of clear guidance in managing such complex cases make treating these infections a real challenge. Of the 76 patients included in our cohort, 54 (71%) did not receive any active empirical antibiotics before the culture results became available. Combination empirical antimicrobials targeted specifically against gram negatives have been used in 29/76 cases (38%), common combinations included carbapenem in addition to amino glycoside sometimes combined with colistin or tigecycline. Piperacillin/tazobactam and carbapenems have been used frequently despite being resistant. On the contrary, new antimicrobials with an excellent susceptibility profile such as ceftazidime-avibactam have been rarely used as an empirical therapy, which is explained by our hospital policy of restricting its use as a targeted therapy with the availability of the susceptibility profile. This, however, has not been associated with a higher hospital or ICU mortality in this study. Wang et al. and others had similar findings [16,24]. 

Piperacillin/tazobactam and carbapenems are frequently used as targeted therapy despite the availability of the sensitivity results; it is not clear, however, whether their use was for treating other sensitive organisms or some clinicians still use carbapenems with either colistin or tigecycline combinations as we have not captured those data. A combination of two antibiotics that CRE is susceptible to was used in only 23.7% of the cohort; while alarmingly 24% have not been on any proper targeted therapy, six patients (8%) did receive antibiotics that were not covering CRE, and 12 patients (16%) did not receive any targeted antibiotic.

Even though monotherapy was reported to be associated with higher mortality compared to antimicrobial combinations [25,26] around half of the patients in our cohort (51%) have been on a single appropriate antibiotic, sometimes combined with other inappropriate antibiotics such as piperacillin/tazobactam or carbapenem. Again, being a retrospective chart review study, we couldn’t draw a firm conclusion as to what was the exact reason for such findings, however, that could be explained by the high resistance pattern of the organisms or lack of involvement of the infectious disease specialists. 

The high all-cause hospital mortality rate of 42% observed in this study was similar to previous reports that showed mortality associated with K. pneumoniae bacteremia harboring KPC ranging from 39 to 82% [24,25]. In another retrospective review of 125 patients with bacteremia due to KPC gene harboring K. pneumoniae, the overall associated mortality rate at 30 days was 42% [26]. That can be explained by the group of patients included in this study, as more than 70% of them are oncology or post-transplantation and more than 60% were admitted to ICU, 34% were diagnosed with septic shock, 28% had bacteremia either primary or secondary and their median hospital stay of around a month.

Similar to other reports, the presence of bacteremia and pneumonia has been shown in this study (Tables 8, 9) to be associated with an increase in both hospital and ICU mortality (P value 0.01 for ICU mortality and 0.04 for in-hospital mortality for bacteremia) (p-value 0.004 for ICU mortality and p-value of < 0.0001 for in-hospital mortality for pneumonia) [16,27]. Part of this excess mortality could be explained by the poor concentration of colistin and aminoglycosides in the lung; given their frequent use to treat CRE infections in this cohort. Tigecycline is also known to be inferior to imipenem in treating ventilator-associated pneumonia [28]. A higher dose of tigecycline might be needed to achieve clinical cure in patients with hospital-acquired pneumonia [29].

Recently (April 2021), the Infectious Disease Society of America published a guideline for the treatment of CRE and MDRO pseudomonas. For CRE management, the recommended first-line antimicrobials are ceftazidime/avibactam or carbapenem-beta-lactamase inhibitor combinations. If metallo-beta-lactamase is present, then the drug of choice will be either ceftazidime/avibactam combined with aztreonam or cefiderocol [30]. Practice change driven by recently published evidence takes time, as those medications become widely available and incorporated into routine practice.

Limitations of the study

Our study is retrospective, single center, mainly descriptive with a small number of patients, lacks a control group, and is not powered to establish causality. Molecular detection of the CRE genes was reported in a small number of patients as this test was introduced in the hospital late in the study period. However, we thought that we should share our data on these emerging and increasingly resistant organisms and assess the likely factors that could be contributory to the infection with those pathogens and describe their treatment and the associated outcomes.

In the last few years some relevant studies have been published from other centers in KSA and other Gulf Countries. However, we think our study is still important as it focused on a particular type of patient as most of our sample included immunosuppressed patients with cancer or transplant given the main focus of our center. In addition, we have described a pattern of antimicrobial utilization among our physicians for both empirical and targeted therapy and we described patient outcome which has not been described in other published trials; this calls for an urgent need for strict policies that drive more attention from the practitioners treating such deadly infections and from policy makers, as the problem will most likely be global. We think this study will serve as evidence in recognizing the problem and pushing forward for research-informed policy making.

## Conclusions

Infections due to CRE are common and their prevalence is increasing. Their attributed mortality is high. Lack of clear guidance and limited treatment options has made a great variability in practice. Enhanced infection control practices, appropriate utilization of antimicrobials with activation of antimicrobial stewardship programs, and availability of more effective antimicrobial drugs are urgently needed to defeat this global health threat. These preliminary observations provide an important platform for larger trials that focus on such infections.
